# "The Hospital" Nursing Mirror

**Published:** 1897-02-13

**Authors:** 


### "The Hospital" Nursing Mirror.

Being the Nursing Section of "The Hospital."

[Contributions for this Section of "The Hospital" should be addressed to the Editor, The Hospital, 28 & 29, Southampton Street, Strand, London, W.C., and should have the word "Nursing" plainly written in left-hand top corner of the envelope.]

### News from the Nursing World.

### QUEEN'S COMMEMORATION AT NORWICH.

The Earl of Leicester presided over a very large meeting held in the Guildhall at Norwich to consider the form in which the sixtieth year of the Queen's reign should be celebrated in Norfolk. He suggested that Her Majesty would be best pleased if the permanent local memorial with which he felt sure Norfolk and Norwich would wish to mark their loyalty and respect took a form which tended in some direction to the relief of the sick and suffering, and the Dean then proposed that a new Jenny Lind Infirmary for Sick Children should be built, to take the place of that established by the great singer many years ago, and now inadequate to the demands upon its accommodation, and for which a site has been presented by Mr. J. J. Colman. The resolution was unanimously agreed to, and a committee appointed to raise the necessary funds. Lord Leicester, on the condition that the hospital should be entirely devoted to the treatment of children, that it should not clash with the Norfolk and Norwich Hospital, nor be larger than the annual subscriptions could maintain, said he would head the list with a donation of £2,000.

### A NURSES' CLUB FOR BOURNEMOUTH.

A scheme is on foot to form a club for nurses at Bournemouth which should prove a distinct success. Bournemouth, more than most places, perhaps, needs some such place of resort for nurses, both those attached to the many institutions in the town and private nurses who accompany their patients to this favourite haunt of the invalid. A meeting was recently held to consider the plan, Miss Christina Forrest, late matron of the York Hospital, and now lady superintendent of the Victoria Nurses' Institute at Bournemouth, in the chair, and Miss Scott, late matron of the Sussex County Hospital, explained the details of the proposed club to the audience. They will include a quiet resting-room, well supplied with papers, writing-tables, &c., a free lending library, lectures on other than professional subjects, social gatherings, and daily afternoon tea. Members' subscriptions, it was suggested, should be 5s. quarterly, 3s. monthly, and 1s. weekly, these latter terms being for the benefit of the nurses temporarily visiting Bournemouth. The scheme is being supported by many matrons of hospitals and homes in the neighbourhood, and besides Miss Forrest and Miss Scott the following ladies will be pleased to hear from those wishing for the formation of the club or to receive suggestions as to its working: Miss Sybil Airy, matron, Victoria Hospital; Miss Maguire, matron, Firs Home; Miss Mellersh, matron, Herbert Home; Miss Milne, matron, Poole Hospital; and Miss White, matron, Boscombe Hospital.

### THE ROYAL NATIONAL PENSION FUND.

The Royal National Pension Fund for Nurses should by this time be thoroughly well known amongst all who interest themselves in the welfare of nurses, but there still seems a want of understanding in many quarters as to its scope and aims. Quite lately someone started a proposal to celebrate the sixtieth year of the Queen's reign by establishing a pension fund for Irish nurses. in apparently blissful unconsciousness of the already existing institution, to which many Irish nurses belong. In the January number of *Asylum News*, too, the Rev. Henry Hawkins, in a letter on the Association of Asylum Workers, suggests that "an Asylum Nurses' Pension Fund" might be instituted "similar to the Royal National Pension Fund for Nurses," or that a plan might be devised to incorporate the funds. Asylum attendants have always been eligible to join the Royal National Pension Fund.

### QUEEN'S NURSES IN SCOTLAND.

The eighth annual report of the Scottish Council of Queen Victoria's Jubilee Institute for Nurses is pleasant reading, showing the steady spread of Queen's nurses over Scotland. Fourteen new branches have been affiliated to the institute during 1896, there now being seventy-two affiliated associations in Scotland and one hundred and twenty-nine Queen's nurses. Miss Wade and Miss Hadden made ninety inspections of the nurses and smaller homes during the year, and were able to give most satisfactory reports of the nurses' work. Of course, the report makes prominent mention of the great event of the past year, the Queen's reception of her nurses at Windsor, to which sixty-nine Scottish nurses went. These nurses were received the next day at Kensington Palace by Princess Louise, Marchioness of Lorne, President of the Scottish Branch. The only regrettable fact mentioned in the report is a falling off of something like £100 in annual subscriptions. Special bequests amounting to £6,500 are reported by the Council, by the help of which, and by other special donations to the building fund, it has been possible to improve and alter the new premises purchased last year and add them to the home.

### ASSOCIATION OF ASYLUM WORKERS.

The Association of Asylum Workers held its second annual meeting on Monday at the Trained Nurses' Club, Buckingham Street. The association, whose first president was the late Sir Benjamin Ward Richardson, has for its chief objects (1) to improve generally the status of asylum nurses and attendants; (2) to secure the sympathy and co-operation of all those interested in institutional work and efforts; and (3) to provide a home of rest and nursing for those engaged in asylum work, no such home being now in existence. On Monday the meeting was but a small one, the business being of a purely formal character. Mr. P. Michelli took the chair, and amongst those present were Dr. Shuttleworth, Dr. Walmsley, Dr. Jenkins, Miss Honour Morten, and Miss Laura Evans, of Berrywood. The report for the past year was adopted. Dr. Shuttleworth was unanimously appointed hon. secretary in place of Dr. Walmsley, who resigned in consequence of pressure of work; the executive committee was elected, and a resolution was passed urging the committee to press forward the institution of the proposed Home of Rest. The association is practically only just starting, but already boasts nearly 2,000 members, and has been warmly welcomed by many medical superintendents of asylums throughout the country. The first number of *Asylum News*, the journal of the society, was published in January of this year, and contains the annual report and financial statement for 1896. For bringing out the paper Dr. Cassidy, of the County Asylum, Lancaster, placed the printing presses of the asylum at the disposal of the association. It was announced at the meeting that all the nurses of the Norwich Borough Asylum had signified their intention to join the association.

### ROYAL BRITISH NURSES' ASSOCIATION.

MISS ADA PRITCHARD has been appointed secretary of the Royal British Nurses' Association. Miss Pritchard has acted as treasurer to the Oxford Women's Liberal-Unionist Association, in connection with which she undertook the hon. secretaries' duties occasionally. She has also assisted in the secretarial work of the Junius S. Morgan Benevolent Fund, and has generally had much experience in organising and in business correspondence. Her testimonials show her to be an able and enthusiastic worker, ready to throw her whole energies into any work she undertakes, and we therefore congratulate both her and the Association on the appointment.

### MR. BANCROFT AND THE HOSPITALS.

ON Tuesday this week Mr. Bancroft gave his reading of "A Christmas Carol" at the Music Hall in Edinburgh, in aid of the Royal Hospital for Children; on Friday repeating it at the Guildhall, Cambridge, on behalf of the Nurses' Institute. Next Tuesday he completes the series of readings at Stafford House, in aid of the Chelsea Hospital for Women.

### CONVENTION OF TRAINING SCHOOL SUPERINTENDENTS.

THE fourth annual meeting of the American Society of Superintendents of Training Schools for Nurses is being held this month in Baltimore. There will be some interesting discussions on nursing matters, papers being read by Miss Isabel Merritt (Brooklyn City Hospital), Miss Kimber (New York City Training School), Miss McKechnie (City Hospital, Wilkes Barre), Miss Walker (Pennsylvania Hospital), and Mrs. Hunter Robb (formerly Miss Hampton), while many other well-known leaders in the nursing world are announced to take part in the discussion.

### THE BELFAST HOSPITAL FOR SICK CHILDREN.

A RECEPTION was lately given at this hospital by the Hon. R. T. O'Neil, President, and Mrs. James McCalmont, Lady President, in honour of the visit of Lord Lister and Lord and Lady Kelvin. The guests, some 300 in number, were received in the nurses' dining-room, prettily decorated for the occasion. The distinguished visitors were taken by the medical staff over the hospital, and expressed themselves pleased with the arrangements, the operating theatre attracting particular notice. This part of the hospital, with the out-patient department, was greatly improved and enlarged about eighteen months ago. Tea and coffee were served in the out-patients' hall, transformed for the time by means of drapery, flowers, and plants, the late chairman of the board, always a very good friend to the hospital, generously sending many handsome palms and other plants to make the place look festive. Lady Londonderry, in going round the wards, was much interested in a little boy who had been operated on for spinal disease. He chatted to her happily, and was very proud of her giving him, as he expressed it, a "sweet kiss." The hospital consists of two wards, 18 cots in each, and there is much out-patient work. At the present time the hospital greatly needs funds, as it is entirely supported by voluntary subscriptions, and these do not cover the expenditure. Many improvements must be postponed owing to lack of means to carry them out.

### AN AMERICAN LADY DISPENSER.

A LADY, who is a trained nurse as well as a qualified dispenser, has recently been appointed to the superintendence of the dispensary at Manhattan State Hospital from the State Civil Service eligible list. Miss Mahony is a graduate of the New York City Training School for Nurses, Blackwell's Island, afterwards studying medicine in the medical ward of St. John's Guild, Staten Island. She then qualified at the New York College of Pharmacy, and started for herself in Brooklyn, giving this up to accept the position of head nurse at an institution on Blackwell's Island.

### NURSING LECTURES IN IRELAND.

AN excellent course of "Homely Talks on Sick Nursing" is being given in the lecture-room of Christ Church, Rathgar. On the last Saturday in January Miss Dunn, superintendent of the Irish branch of the Queen's Jubilee Institute, gave instruction on the important subject of "How to Make a Good Poultice," and for the following Wednesdays and Saturdays Miss Lee, matron of the National Ear and Eye Infirmary, Dublin, Miss Fitzpatrick, matron of the Adelaide Hospital, and Miss Huxley, matron of Sir Patrick Dun's Hospital, promised their services.

### A NURSE FOR ST. IVES.

A PUBLIC meeting was held lately at the St. Ives Town Hall, under the presidency of the Mayor, to consider the need for establishing a district nurse for the town and its near neighbourhood. It was decided to form a committee with the object of ascertaining whether funds for the purpose could be raised.

### COLONIAL NURSING.

EXCELLENT reports are received by the Colonial Nursing Association of the two nurses who have been working in Mauritius from the first starting of the association. Nurse Cunnington and Nurse Bothwell have been doing valuable service, and "won golden opinions" from all with whom they have come in contact. It is stated that a movement is now on foot in the colony, with the co-operation of the Government, to enable the nurses to use their spare time in training probationers in the Government Hospital, where until now trained nursing has been a thing unknown.

### TO CORRESPONDENTS

WE have received many letters on subjects of current interest which for want of space must be held over till next week. We take this opportunity of reminding our readers once more that letters for publication in "this week's" HOSPITAL should reach the office not later than Monday morning. Also let us once more impress upon correspondents the excellent maxim that "Brevity is the soul of wit," and that no letters should exceed three to four hundred words in length.

### Pharmacy and Dispensing for Nurses.

By C. J. S. Thompson.

III.—ANTISEPTICS—ANTISPASMODICS—ASTRINGENTS—CARMINATIVES—CATHARTICS—CAUSTICS—DEMULCENTS—DIAPHORETICS—DISINFECTANTS—DESICCANTS.

ANTISEPTICS are agents which prevent the decomposition of organic structures, and therefore of great value in surgery. This class includes a large and increasing number of chemical bodies, the majority of which it will be only necessary to enumerate. The following are those chiefly employed: Acids: benzoic, boric (used as a dressing for granulating and suppurating surfaces in general, also in lotions and eye-washes, &c.), carbolic (as a dressing, with water or oil 1 in 20 or 1 in 40), chromic, cresylic, hydrochloric, nitric, pyrogallic, salicylic (in solution as a preservative), sulphurous, trichloracitic, acetate of alum, aristol, airol, alum solution, alumnol, alum chloride solution, alum oleate, ammonium carbonate, antifebrin, antipyrine, borax, boroglyceride, chlorinated lime, charcoal, bisulphide of carbon, chinolin, cream of tartar, chlorine solution, chloroform, creasote, copper oleate, eucalyptol, europhen, glycerin, helenin, mercuric cyanide, mercuric zinco-cyanide, mercuric perchloride (in solution with water 1 in 1,000, a powerful antiseptic and bactericide), mercury and potassium iodide, iodoform, iodol, iodine, loretin, lysol, menthol, napthalene, napthol, nosophen, sulphurated potass, phenosalyl, permanganate of potass, resorcin, sal alembroth (used in the antiseptic treatment of wounds, in the form of solution, gauze 1 per cent., or wool 2 per cent.), salol, chlorinated soda solution, benzoate of soda, chloride of sodium, sodium sulphite, sodium sulphocarbolate, sozoiodol, turpentine oil, thalline sulphate, thymol, tribromophenol, trichlorphenol, zinc chloride, and zinc sulphocarbolate.

ANTISPASMODICS are medicines which allay or prevent the recurrence of spasms. They include acid hydrocyanic, ammonia, ammon. carbonate, aromatic spirit of ammonia, ammoniacum, amyl nitrite, nitrate of silver, asafœtida, belladonna, bromides, calendula, camphor, Indian hemp, cerium oxalate, chloral hydrate, chloroform, cimicifuga, conium, ether, galbanum, grindelia, henbane, lobelia (chiefly used in spasmodic asthma), musk (used in hysteria), oil of peppermint, nitro-glycerine (used in solution and in tablets, chiefly for angina pectoris), quebrache, rue, stramonium, sumbul, fetid spirit of ammonia, turpentine, valerian and the valerianates (used in hysteria), and zinc sulphate and oxide.

ASTRINGENTS are another large class of remedies which produce contraction of the tissues and coagulation of the albuminous fluids. They are given to improve digestion and check increased secretions, mucous discharges, and hæmorrhages, or applied locally to prevent relaxation and to stop bleeding. The principal mineral bodies used as astringents include all the diluted mineral acids, alum, nitrate of silver, oxide of silver, borax, cadmium sulphate, calamine, calcium carbonate, carbolic acid, creasote, prepared chalk, copper sulphate, iron perchloride, iron pernitrate solution, iron sulphate, iron and quinine citrate, lead acetate, carbonate, and oxide; solution of subacetate of lead, zinc acetate, carbonate, chloride, oxide, sulphate and sulphocarbolate. The vegetable substances include acetic acid diluted, gallic acid (given in all cases where the bleeding vessels must be reached through the circulation), tannic acid (useful when the mucous membrane is relaxed, bleeding at nose and uterine hæmorrhage), bael fruit (employed in dysentery and diarrhœa), catechu (chiefly used in diarrhœa), cinchona, cinnamon, cornutine, ergot (valuable in uterine hæmorrhage), male fern, pomegranate root, guarana, red gum, logwood (used in diarrhœa), hamemelis, hydrastis, rhatany, kino, larch, matico, oak, roses, rumicin, turpentine, elm, bearberry, vinca major, and vinegar.

CARMINATIVES are medicinal agents which stimulate or aid the removal of flatus from the stomach and intestines, and relieve griping. Nearly all the aromatic essential oils are used for this purpose, including dill, anise, carraway, cinnamon, coriander, fennel, juniper, lavender, lemon, mint, peppermint, and nutmeg. Acetic ether, camphor, charcoal, laudanum, pepper and ginger are also largely employed as carminatives.

CATHARTICS are medicines which promote intestinal evacuations or induce action of the bowels. They may be divided into three classes, viz: 1. Mild or laxative, include belladonna, cassia, euonymin, ox gall, figs, liquorice powder (compound), ipecacuanha, magnesia and its carbonate, manna, mulberry juice, nux vomica, olive oil, potassium sulphate and tartrate, prunes, rhamnus frangala, cascara sagrada, castor oil, soap, effervescing citro-tartrate of soda, phosphate of soda, sulphate of soda, tartarated soda, sulphur, precipitated sulphur, tamarinds, dandelion, and treacle. 2. Active aperients: Aloes barbadoes and socotrine, bapterin, colchicum, black hellebore, iridin, kamala, leptandrin, sulphate of magnesia (Epsom salts), podophyllin, rhubarb, and senna. 3. Strong purgatives: Bryonia, gamboge, colocynth, croton oil, elaterium, black hellebore, calomel, jalap, acid tartrate of potassium, and scammonium.

CAUSTICS are substances which destroy the vitality of the parts to which they are applied. They include: Glacial acetic acid (largely employed for destroying corns and warts), arsenious acid (used in the form of paste to destroy the sensibility of carious teeth), acid carbolic, acid chromic (a powerful agent which will readily dissolve all animal tissues), acid nitric, acid sulphuric, chloride of antimony, nitrate of silver (for cauterising poisoned wounds), lime, creasote, acetate and sub-acetate of copper, nitrate and sulphate of copper, red iodide of mercury, red oxide of mercury, perchloride of mercury, acid nitrate of mercury, iodine liniment, caustic potash, permanganate of potass, caustic soda, solution of ethylate of sodium (used in the treatment of nasal polypus and lupus), chloride and nitrate of zinc.

DEMULCENTS are substances which soften and allay irritation of mucous membranes. The following are chiefly employed: Gum acacia, marshmallow, sweet almond, starch, spermaceti, Irish and Iceland moss, quince seed, figs, glycerin, liquorice, barley, isinglass, linseed oil, arrowroot, honey, cod liver oil, olive oil, albumen of egg, treacle, tragacanth, creeping couch grass, elm bark, and raisins.

DIAPHORETICS are medicines which increase the action of the skin and promote sweating. They are given in case of chill, also in fevers, dropsy, and some skin diseases. They include ether, alcohol, solution of acetate of ammonium, ammonium carbonate, chloride, and phosphate; antimonial powder, tartared antimony, wine and sulphurated antimony, horse radish, buchu, spirit of cajapute, calendula, camphor, chloroform, wine of colchicum, Dover's powder, dulcamara, grindelia, ammonicated tincture of guaiacum, ipecacuanha powder and wine, jaborandi, lettuce, lobelia, morphine, opium, pilocarpine, potassium citrate and nitrate, savin, sassafras, senega, simarubra, serpentaria, spirit of nitrous ether, sulphur, and oil of turpentine.

DISINFECTANTS are substances which act on the specific poisons of communicable diseases so as to prevent their spreading. Amongst these in general use are acid carbolic, acid nitrous, acid sulphurous, solution of chloride of alum, chlorinated lime, chlorine, iodoform, iodol, iodine, eucalyptus oil and preparations of the leaves, naphthol, permanganate of potass, Condy's fluid, potassium bicromate, sulphate of iron, hydrogen peroxide, sulphur dioxide, chlorinated soda solution, terebene, thymol, trichlorphenol, zinc chloride in solution (known as Burnett's solution); also the numerous carbolic and coal tar derivatives sold under various names. The majority of disinfectants also act as deodorisers and destroy offensive smells.

Desiccants are agents which check secretion and dry up mucous discharges from ulcers and wounds. They include bismuth subnitrate, calcium carbonate, calcium hydrate, prepared chalk (used externally for burns and ulcers), magnesium carbonate, lead acetate and carbonate (employed in diminishing profuse discharges from ulcers), talc, and zinc oxide.

### Post-Graduate Clinics for Nurses.

By a Trained Nurse.

### II.—THE EVOLUTION OF THE PRIVATE NURSE.

A nurse may make an admirable "staff," she may understand to perfection the management and discipline of a ward, and, in addition, be well skilled in her art. But, put in charge of a patient in a private house, she may prove a complete failure. The qualities needed in a hospital worker differ so essentially from those which are acceptable and necessary in a nurse undertaking the care of the sick in their own homes. Despite the enormous development in the art of nursing and the efficiency our Training Schools have attained during the past twenty years, much remains to be done in order that the curriculum of hospital training shall be made more satisfactory and complete. A nurse who has finished her three years' training and obtained her certificate—perhaps in the best Training School extant—dreads with a painful nervousness the ordeal of her "first case" in a private house. And the dread is well founded, for, speaking generally, the training in our English hospitals has taught her nothing that she needs to know of the nursing of good class and educated patients in their own houses. The discipline which she has been taught to consider as the first element towards her success as a staff nurse is the very last element which will make her popular and successful in private practice. The scrupulous order and neatness of a hospital ward must be exchanged in private houses for the more or less artistic confusion so dear to the heart of the home invalid; while the wholesale and somewhat rough-and-ready style in which the diet of hospital patients is administered offers no training in the dainty service of food which private patients expect. Among the common stumbling-blocks of the hospital nurse emerging into private work a love of discipline and a rigid maintenance of order are, perhaps, those which cause her the most anxiety.

She emerges from the Training School armed with certificates and testimonials setting forth her admirable qualities as a ward worker and her multiple excellences in dealing with the sick. But this same nurse, confronted, perhaps, as her first patient with a society woman accustomed to the ministrations and nice "fussiness" of a skilful lady's maid, will be apt to realise that there is more art in private nursing than was dreamt of in the philosophy of her Training School.

"My nurse gives me no peace in her everlasting endeavour to keep my bed tidy" is a universal complaint of patients against the enthusiastic nurse new to private work, who does not realise that the symmetry essential to a long row of ward beds is a different matter from the one bed of a restless private patient who has not been accustomed to be scolded and held responsible for the slipping to one side of her eider-down quilt or elaborate bed-spread.

"My nurse treated me as I should think a prison warder would treat a convict," said a lady in criticism of a nurse fresh from the Training School, who had tried to introduce into a private room containing one patient, educated and refined, the rules and regulations necessary to keep in order a ward holding some twenty or more persons untrained, and often of an uneducated and unruly calibre.

"She actually wanted to cut my hair short when she first came," continued the horrified patient, "because my sister, who looked after me before the nurse came, let it get in a tangle. Just imagine shearing me like a charity school girl," she concluded, indignant at a suggestion which under very tangled and unclean conditions in a hospital patient might have been necessary, but which was absolutely unsuitable to put into practice in the case of a lady whose pretty dainty hair was one of her most valued considerations! The nurse in question in learning how to untangle the hair learnt at the same time a very valuable lesson in the ethics of private nursing, and she is most successful in her work now that she has grasped the essential difference between patients at home and in hospital.

Supposing the nurse fresh from the Training School to belong to the cultured classes, to be herself a woman of refinement and accustomed to dainty surroundings in her own home, it can hardly be denied that the years she has spent in the wards of a hospital will by no means have increased her æsthetic sense of the surroundings of the sick. Institution life is plain and bare; it is based on rule and regulation, and there is little time for "fadding" and humouring the whims of individual patients. Everything is done for the sick that is kind and considerate, and the nurse shows an unselfishness and devotion which could not be exceeded in private work, but the rose-coloured glow, and the little fripperies and perfumeries and refinements which permeate the private sick-room are necessarily absent from a ward. And at present there is no compromise—no intermediate stage of training between the care of the sick coal-heaver and the cabinet minister, the laundress and the duchess. I must apologise to my readers for the comparisons between English and American hospital methods which I must introduce somewhat frequently into these "Clinics." But such comparison is not intended in an invidious sense. Both systems have their faults and drawbacks. And it must not be supposed when I draw attention to the many excellences obtaining in American hospitals that I am in any sense depreciating our own. But it seems most helpful to contrast various systems; and it is only by comparison, the adoption of the good and the rejection of the bad, that we shall be able to reach the highest ideal and standard in our Institutions. So far as a preparation in her Training School for the duties of private nursing goes, the American hospital offers many advantages.

First and foremost among the advantages, so far as the nurse is concerned, may be counted that system by which most of the hospitals in the United States set aside a number of rooms for the reception of private paying patients. It is usual for each nurse during her training to serve so many months in attendance on these paying patients, so that she receives in the ordinary routine of her work an insight into the method of caring for the rich, the "faddy" and luxurious, and the many varieties of type that are to be found in the range of private practice. So that if she elect, on the completion of her training, to take up private nursing as a career, she is already equipped for the work, confident and trained for that which she has to do. Even in those Hospitals—always excepting the "charity" Hospitals such as Bellevue in New York and Blockley in Philadelphia, where the class of patient corresponds to those in our Poor Law Infirmaries—where there are no private rooms, a large percentage of American patients pay something towards "board and nursing," and exact as a consequence a standard of personal care and consideration in the matter of hair-dressing and the arrangement of frilled nightgowns, &c., as would mightily astonish the nurses of our London hospitals, accustomed rather to unwillingness on the part of their patients to submit to necessary washing and dressing than to complaints of insufficient toilet attention, which any remissness on the part of the nurse would soon call forth from the paying patient of an American hospital.

### Nursing in New South Wales.

### PRINCE ALFRED HOSPITAL TRAINING SCHOOL FOR NURSES, SYDNEY.

THE excellence of the training which is given to nurses at the Prince Alfred Hospital, Sydney, is matter of general knowledge. English nurses are sure, therefore, to be interested in seeing for themselves the questions set in the recent annual examinations at this training school, which we give below:—

### FIRST YEAR CANDIDATES.

ANATOMY AND PHYSIOLOGY.—1. Of what is bone composed? 2. What are the following: (*a*) Ligament; (*b*) tendon; (*c*) periosteum; (*d*) red marrow. 3. Tell what you know of the functions of the pancreas. 4. How does blood get from the left auricle into the right ventricle? 5. State briefly the positions of the subclavian artery, the femoral artery.

INVALID COOKERY.—1. How would you make one quart of good beef tea? 2. Which fish would you choose for an invalid? Give one recipe for dressing it. 3. Give recipe for a milk pudding. 4. How would you boil mutton and prepare paisley sauce for an invalid? 5. Describe how you would cook a suitable vegetable for an invalid. 6. How would you make a restorative broth or strengthening soup? 7. How would you cook an apple and make a boiled custard?

ELEMENTARY NURSING.—1. A new patient is admitted to the ward, state fully your treatment of the patient till the doctor arrives. 2. Mention some of the different kinds of enemata. How would you prepare and administer a nutrient enema? 3. You are required to sponge a patient suffering from typhoid fever, state briefly how it should be done. 4. How would you give the following?—(*a*) Hot air bath; (*b*) Vapour bath. 5. Give your method of nursing a patient with paralysis.

### SECOND YEAR CANDIDATES.

MEDICAL NURSING.—1. Enumerate the principal points to be noted in taking a patient's pulse, and how would you ascertain each? 2. Define, do not describe: (*a*) Syncope; (*b*) Rigor. State what immediate treatment you would adopt in the case of each. 3. In the absence of the medical attendant or while awaiting his arrival, what means of treatment may the nurse adopt in case of (*a*) Vomiting; (*b*) Diarrhœa? 4. Briefly mention the chief points to be attended to in nursing a patient in the "typhoid state." 5. In the event of nursing a case of scarlet fever in a private house, what precautions would you take to prevent the infection of others both during the patient's illness and after his recovery?

NURSING SICK CHILDREN.—1. How would you prepare beef juice and bread jelly? 2. Tell what you know of ulcerative stomatitis; how would you treat it? 3. What is an intussusception, and what are its symptoms? 4. How would you artificially feed a child three months' old?

MATERIA MEDICA.—1. What do you understand by the terms—Materia medica, decoction, therapeutic, lotion, anodyne, disinfectant? 2. What circumstances modify the action of drugs in their administration as medicines? 3. What are alkaloids? What advantages do they possess over other preparations? 4. How do antipyretics lower a patient's temperature? What are the more commonly used antipyretics? When are they best administered, and what dangers attend their administration? 5. What are the symptoms of a poisonous dose of morphia? Sketch the treatment of a case of opium poisoning.

SURGICAL NURSING.—1. Describe the action of, and the changes which occur in, lymphatic vessels and glands in a case of acute inflammation, say of the hand and forearm. 2. Mention the different methods by which wounds may heal. Describe the method of "healing by granulation." 3. Give the meaning of the following terms: (*a*) Septic; (*b*) aseptic; (*c*) antiseptic. Describe in detail the preparations you would make before commencing to dress a wound antiseptically. 4. A patient has had a severe internal hæmorrhage caused by an injury to the abdomen without any external wound. What symptoms would be present and how would you nurse him? 5. How would you prepare a patient for the operation of "Trephining the skull?"

### THIRD YEAR CANDIDATES.

MASSAGE AND ELECTRICITY.—1. How would you perform effleuage on the lower limbs? Define the terms centripetal and centrifugal. 2. The effects of massage may be arranged under four heads, name and briefly describe them? 3. How would you treat a case of constipation by massage? Or, describe your mode of procedure in the massage of a joint. 4. What are the three principal forms of electricity? Briefly describe faradism. 5. Describe how you would test electrical reaction by the polar method. 6. Define electrolysis, and name a case in which it may be applied.

OPHTHALMIC NURSING.—1. How would you tell conjunctival from ciliary congestion? What is the importance of distinguishing between the two? 2. Give a list of the ordinary lotions and drops used in ophthalmic work, with the strength of each, under the following heads: (*a*) Antiseptic, (*b*) astringent, (*c*) stimulating, (*d*) sedative, (*e*) mydriatic, (*f*) myotic. 3. What dangers, local and general, attend the use of atropine? How would you recognise these dangers from the symptoms? How guard against them?

NURSING OF THE INSANE.—1. What is a delusion? And an hallucination? Give an example of each. 2. What is melancholia? In nursing a case of melancholia what points must you specially attend to? 3. What forms of insanity are associated with child-bearing? Describe briefly a case of puerperal mania. 4. What is delirium tremens? In what cases is it likely to occur in a general hospital? 5. In selecting and preparing a room or suite of rooms for a probably suicidal patient, what points would you specially attend to?

GYNÆCOLOGICAL NURSING.—1. How would you know a patient was suffering from hæmorrhage after abdominal section, supposing no drain had been used? 2. Prepare a patient for vaginal hysterectomy. 3. How would you clean a set of abdominal sponges? 4. What symptoms would induce you to send for the surgeon within eighteen hours after an abdominal operation? How would you send the message?

### Nursing in India.

### THE LADY KINNAIRD HOSPITAL, LUCKNOW.

A NEW wing was opened at the Lady Kinnaird Hospital, Lucknow, towards the close of last year, increasing the number of beds by twenty-six, and adding bath-rooms and offices. The new building is divided into two large wards with eight beds in each, three small wards, each containing two beds, and four in the isolation ward. White markeen curtains, bordered with red, divide the large wards up into cubicles when required, each patient having a small table, a locker, and a bracket for medicines beside the bed. The wards are surrounded with verandahs, pleasant places for the convalescents to sit in. On the day of the opening, the ceremony being performed by Mrs. Deas, wife of the Judicial Commissioner, the isolation ward was arranged as a tea-room, and made pretty with plants and flowers, and the verandahs filled with lounges and easy-chairs for the guests.

The history of the Kinnaird Hospital shows how increasingly the people of India avail themselves of the medical aid offered to them. It was in 1876 that the Zenana Mission first opened a dispensary for female patients in Lucknow, and the work growing under the care of Drs. Alice Marston, Izset Mead, and Jane Haskew so that the accommodation became all too small for the demands upon it, in January, 1891, the foundation of the present hospital was laid by Sir Auckland Colvin, the building being opened in October of the same year by the Hon. Gertrude Kinnaird. The hospital was dedicated to the memory of the late Lady Kinnaird, whose interest in the welfare of the women of India was unflagging throughout her life. The building contained 24 beds, quarters for the matron and nurses, a dispensary, and a bungalow for the lady doctors. In 1895 the number of out-patients treated at the hospital during the year had reached 14,705. From a nursing point of view the hospital is also proving most useful, Miss Sheldon, the English matron, carrying on the work of training native Christian girls as nurses. They are intelligent and teachable pupils. Miss Jane Haskew, Miss Lilian Jenkins, and Miss Cornall are the ladies on the medical staff of the hospital.

### The Victoria Club for Nurses.

We announced last week the establishment of a club for nurses and associated workers, and explained its objects and gave some details as to its arrangements. On Thursday, the 4th inst., the club was thrown open for inspection, and in response to an invitation from the trustees of the Hospital Building, in which it is situated, Miss Thomson, the secretary, received a large and representative number of visitors. As early as nine o'clock in the morning of Thursday some of the nurses were welcomed, but the majority of guests arrived between four and six o'clock in the afternoon. Miss Thomson was indefatigable, and she and her little band of assistants were kept well employed explaining the arrangements and answering questions. Miss Thomson must have been gratified by the unanimous and hearty admiration expressed by the visitors. Each room in turn was pronounced to be as complete and charming as possible. The building lends itself exceptionally well to the purposes of such an establishment. The ample window space, and quaint turret corners, give an air of freshness and originality which it is not easy to find in London habitations. The little frilled silk window curtains or blinds are especially delightful, and the scheme of colour which prevails is artistic and harmonious; the soft yellows, browns, and stone colour of the decorations affording a pleasing background to beautiful Egyptian rugs, with which the rooms are carpeted throughout. These and many other beautiful specimens of Egyptian handicraft are Miss Thomson's own property, and were brought by her from Cairo. A number of treasures are to be found in the secretary's office—a most cosy apartment, filled with pretty things. If the luxurious drawing and tea-rooms gave anyone the impression that comfort and ease alone were the objects of the establishment, such thoughts were banished when the excellently-appointed writing and lecture-rooms were visited, and the business arrangements found to be equally complete. The nicely-fitted lavatories on each floor, the cloak-room with its pretty case of toilet necessaries, and the clean, bright, and roomy kitchen at the top of the building, appealed especially to the domestic-minded; and, in fact, the whole establishment received the admiration it certainly merits, and several guests unattached to nursing or institutional life, save through interest, expressed regret that so charming a club was not open to them. We heard several private nurses remarking that there was no need now to be dependent on the dingy sitting-room of furnished apartments if you had a lovely place like this to read, write, or rest in, and we heard a nurse inquiring for a bedroom near the club. The many who visited the club on Thursday felt that they were amply repaid for their temerity in venturing out in spite of the inclement weather, and numbers expressed regret that they were unable to come. Hospitality was dispensed with lavish hand, and the tea-room looked especially inviting as the guests clustered round the snowy-covered little tables, gay with mimosa and daffodils and covered with pretty china and tempting dainties. It was the desire of the trustees that all nurses should be made welcome, and invitation cards were sent to the matrons and nurses of all the London and suburban hospitals, infirmaries, district nursing centres, and private nursing establishments, without limitation as to numbers. Amongst those also invited were the Director-General of the Army Medical Department and the army and navy sisters. We noticed also his Eminence Cardinal Vaughan moving with kindly interest from room to room in company with many not directly associated with the objects of the club. Doubtless those present have inspired their friends with a wish to see the pretty little club, and so we have obtained the secretary's permission to state that she will be pleased to show the establishment to any nurses who would like to see it at any time they can manage to come. Miss Thomson knows what busy people nurses are, and so dispenses with mentioning the "fixed hours," which are so difficult for nurses to fall in with. She informs us that application for membership are coming in daily, and so she asks us to remind nurses that there is no entrance fee until June next, after which time the entrance fee will be £1 1s. Associated workers will be required to pay the entrance fee from the present date.

### Everybody's Opinion.

[Correspondence on all subjects is invited, but we cannot in any way be responsible for the opinions expressed by our correspondents. No communication can be entertained if the name and address of the correspondent is not given, or unless one side of the paper only is written on.]

### LADY PRIESTLEY AND THE NURSES.

Miss Susan E. Antrobus writes: I think Lady Priestley cannot have an intimate knowledge of foreign hospitals and nursing in general. France, Belgium, and Italy are the countries out of England I know best, and where the nursing is almost entirely in the hands of religious orders. The actual nursing is done by the commonest of the people, generally men or women whose mental powers prevent them earning a livelihood outside the hospital walls. The sisters, who have positively no training, walk about the wards and keep order among their incapable assistants. It follows that there are no appliances such as we are used to. I once took an unconscious woman to a hospital, and there was no carrying-chair. I remarked to the house surgeon that we had one in every hospital. He had never seen one, but had heard that the English were so delicate they needed many more helps than his robuster nation! In a paying hospital I begged for a feeder for a poor lady too weak to sit up; none of the nurses had ever heard of such an article. When a sister is sent for to a private case she sits by the bedside knitting, and at regular intervals administers the medicines. If the patient needs more nursing, she calls in the housemaid. Neither in hospital nor in private houses do they sit up at night. I once remarked to a French sister that in England more sick died by night than by day. "So they do with us," she replied. Then, I said, how could they do without night nurses? Her quiet answer was, "We find them dead when we come on duty." The truth is that none of the nations I have spoken of can bear illness or death, and a nurse's life is only embraced as a mortification in certain religious orders, and by those under them, because no other way of living seems open to them. Of course there is some truth in Lady Priestley's indictment. There are, unfortunately, silly, flighty girls who join the nursing ranks, and it would be well if means could be devised for weeding them out, but the bulk of English nurses are heart-whole in their devotion to suffering humanity, and are a glory to our dear old country.

### THREE YEARS' TRAINING.

"C. H.' writes: You know, of course, that the rules of most nurses' co-operation associations and most schemes for the registration of nurses stipulate that applicants must have had three years' consecutive training at a general hospital. This rule seems likely to act with hardship and injustice on a certain class of nurse, the more so if the idea of second grade nurses, now mooted, ever comes into practice. In order to make my meaning clear, allow me to state a case. A certain nurse at the outset of her career had a small amount of capital, which she determined to expend in getting the most efficient training possible. To that end she paid a fee and received two years' training at a general hospital (of which matrons and others in charge of institutions often say, "A year at that hospital is equal to two anywhere else"). At the end of her two years she went to another hospital for six months' special training in fever nursing, to another for six months of mental nursing, and another for six months of midwifery, making three and a half years' training in all. Yet, on applying at a nurses' co-operation association, the nurse in question was refused on the ground that she had not received three years' training at one hospital. Now a nurse trained for three years at a smaller hospital, where no fee is paid, and where the first year is often more or less spent in "scrubbing," is quite eligible, and would be accepted. And though the nurse whose case I have given would be obviously the better trained, at one association she was told she had no right to expect anything but "guinea" cases. As there must be a considerable body of such nurses, and as the rules in question cannot surely be meant to exclude them, I thought it might be of some use if the hardship entailed by such a rule were pointed out. There can be no reason, I suppose, why it should not be amended to "three years' training in a general hospital or its equivalent."

### THE R.B.N.A. AND MENTAL NURSES.

"AN OUTSIDER" writes: It seems to me, that as it requires three years' training to become an efficient hospital nurse, and scarcely more than two years to become a mental nurse, that it is not fair to the former to place the latter on an equality with them. Nurse Jeannie Duddie writes: "As a rule, three ordinary hospital-trained nurses cannot manage a mental case half as well as one thoroughly trained mental nurse." If this be true it is only natural to suppose that a considerable portion of the training time of the mental nurse is taken up in acquiring this wonderful art or "tact" which surpasses the powers of "three ordinary hospital-trained nurses," and she, therefore, has still less time for her medical and surgical studies. Some mental nurses contend that mental cases are rarely also surgical or medical ones. If this be true, how very limited must be the medical and surgical experience of the asylum-trained nurse? Other mental nurses contend that an asylum infirmary offers the same means of experience as a hospital. If this be true, why is the mental nurse expected to acquire the wonderful "tact" as well as the same medical and surgical knowledge of the hospital nurse in a shorter time. Is the time spent on learning the so-called wardmaid work useless or useful? Should a good nurse consider any duty in a sick room *infra dig.?* Surely in a case where absolute quiet is needed a nurse should willingly undertake to keep the sick room in order. She should do the same in infectious diseases, to avoid the necessity for others incurring the risk of contagion. It is not likely that a nurse who has not done this "ward-maid work" will be likely to be either willing or efficient in a case where her services would be of advantage to the patient or others. Considering the difficult work of the mental nurse, I should think she required an extra year's training before she is as good a nurse for the insane as the hospital nurse is for the sane. When the mental nurse knows as much in surgical and medical matters as her sister, with the addition of having acquired the necessary "tact," then she should not merely be treated as an equal, but as the superior of the hospital nurse. Let all nurses qualify for the R.B.N.A., and mental nurses add the acquirement of the wonderful "tact" which, if we are to credit the words of Nurse J. Duddie, is so woefully deficient in the average hospital nurse.

MISS SOPHIA WINGFIELD, 6, The Orchard, Bedford Park, writes: As I have taken a somewhat active part in the matter now under discussion by the members of the Royal British Nurses' Association, namely, whether or no persons who have not been trained in a general hospital ward shall be admitted as members to our Association, and enrolled upon the Register of Trained Nurses, I would crave your courtesy to reply to a letter by Dr. Outterson Wood which appeared in your last issue. Firstly, Dr. Wood writes: "I claim that all qualified nurses, medical or mental, surgical or obstetric, or fever, or any other class, have 'a right of entry' to the published Register of the R.B.N.A." Here is the whole case in a nutshell. Dr. Wood contends that men and women who have gained experience in the nursing of one branch of disease only are "qualified" nurses. Now, I and those who agree with me contend that no nurse is "qualified" who has not received experience in the nursing of general diseases first, and in the nursing of specialities, such as mental, obstetric, fever, or any other class, second. What Dr. Wood proposes is to degrade our Register of Trained Nurses into a directory of persons who have worked in special hospitals for the sick and asylums for the insane, so that, instead of being a guarantee to the public that each nurse enrolled is thoroughly trained, it will merely be a list of persons who have some knowledge of the nursing of special diseases. Again, Dr. Wood is well aware that he is making a misstatement when he writes: "I am confident it is not the hospital nurses themselves who resist the claims of mental nurses, it is the narrow-minded policy of a few who would restrict the aims and scope of the R.B.N.A." Dr. Wood was present at the last general council meeting, when Dr. Fardon, the hon. secretary, announced that eighteen letters had been addressed to the members of the General Council from nurse members, opposing the admission of specialists, and that only one letter had been addressed to that body supporting the suggestion. Moreover, he is well aware that in answer to 300 letters addressed by me to my nurse colleagues concerning the question, 215 answered protesting against the scheme, and six only giving a tentative consent. These replies from nurses whom I do not know personally is overwhelming evidence that the unbiassed matrons and "nurses themselves" are bitterly opposed to the depreciation of their register. There is very little doubt that if this most deplorable measure is thrust upon us the best class nurses will remove their names from the Register of the R.B.N.A.

### Appointments.

### MATRONS.

CORNWALL COUNTY ASYLUM, BODMIN.—Miss Hope has been appointed Matron at this asylum. She received her training at the Royal Edinburgh Asylum, Morningside, and Harpenden Hall, St. Albans, subsequently being appointed matron at the latter institution.

ELTHAM COTTAGE HOSPITAL.—Miss Amy E. Coke has been appointed Matron of the Eltham Cottage Hospital. Miss Coke was trained at St. Bartholomew's Hospital, and at Queen Charlotte's. She afterwards was appointed successively sister and night superintendent at King's College Hospital, and for the past nine years has been matron at the Royal Infirmary, Windsor.

WYE HOUSE, BUXTON.—Miss Mary Poulton has been appointed to the Matronship of this private asylum. Miss Poulton was trained at the Derby Royal Infirmary, where she afterwards held the position of ward sister, and for two years worked as sister at the West Norfolk and Lynn Hospital. Miss Poulton was recently matron at the Throat and Ear Hospital, Brighton.

PRINCE ALFRED HOSPITAL, SYDNEY, NEW SOUTH WALES.—Two members of the nursing staff at this hospital have recently left to take up other and important nursing appointments. Miss McEwen has been appointed Matron to the Mudgee Hospital, and Sister Dalrymple to the Matronship of the Manley Hospital, a newly-built institution, beautifully situated on the top of a hill overlooking the sea. Nurses Webster and McLean have also left on being appointed staff nurses at the Women's Hospital, Melbourne.

ESSEX AND COLCHESTER HOSPITAL.—Miss Elizabeth Davies, assistant matron at the Chelsea Infirmary, has been successful out of nearly 80 competitors in obtaining the post of Matron at the Essex and Colchester Hospital. Miss Davies was trained at the Chelsea Infirmary, having passed with marked ability through the grades of a probationer, staff nurse, charge-nurse, and night superintendent to the position she now occupies. She will be greatly missed by everyone at Chelsea, where her pleasant manners and kindly disposition have rendered her deservedly popular.

RUSHINGTON HOME FOR WORKING MEN, LITTLEHAMPTON.—Miss Frances Parkes has been appointed to the Matronship of this institution. Miss Parkes was trained at Guy's Hospital, afterwards being appointed first assistant matron and then matron at the Sister Dora Hospital, Milford, Stafford, where she remained for four years. Subsequently Miss Parkes did temporary duty as night superintendent at the Royal National Hospital, Ventnor, was night superintendent at the Derbyshire Royal Infirmary, and charge-nurse at the Eastern Fever Hospital, Homerton. She holds very satisfactory testimonials, and many good wishes for success will follow her to her new post from those amongst whom she has worked.

### Minor Appointments.

Chelsea Infirmary.—Miss Barbara Mackay has been appointed Assistant Matron at Chelsea Infirmary in the place of Miss Davies, who has obtained the matronship of the Essex and Colchester Hospital. Miss Mackay was for two years at the Throat Hospital, Golden Square, and for three and a half years has been at Chelsea Infirmary as probationer and charge nurse.—Miss Winifred Checksfield, who was trained at the Evelina Hospital and has been staff nurse for some time at Chelsea, has been promoted to the post left vacant by Miss Mackay's appointment.

Mill Road Infirmary, Liverpool.—Miss Jessie S. Kember has been appointed Assistant Matron at this infirmary. Miss Kember was trained at the Marylebone Infirmary, where she afterwards held the position of charge nurse. Subsequently she was appointed successively night superintendent at the M. A. B. Hospital, Homerton, and second assistant matron at Poplar and Stepney Sick Asylum, Bromley.

Central London Sick Asylum.—Miss Florence L. Gregory has been appointed Charge Day Nurse at this institution. Miss Gregory was trained at the Lambeth Infirmary, subsequently being promoted to the position of staff nurse.

Blackburn Union Infirmary.—Miss Ellen Bumstead has been elected to the post of Superintendent Nurse at this infirmary. She was trained at King's College Hospital, afterwards being appointed nurse at the Doncaster Union Workhouse Infirmary.

### Where to Go.

The Nurses' Co-operation.—The ordinary general meeting of The Nurses' Co-operation will be held at 8, New Cavendish Street, on Tuesday, February 16th, 1897, at 4.30 p.m.

Royal British Nurses' Association.—The third sessional lecture of the season will be given on Friday, February 26th, at eight p.m., at the offices of the Royal British Nurses' Association, 17, Old Cavendish Street, W., by Dr. Henry Kenwood, the subject being "Home Sanitation," with lantern illustrations. Members are admitted free, the general public on payment of 1s.

Kensington Town Hall.—A grand bazaar in aid of the Church of England Home for Waifs and Strays will be held at the Kensington Town Hall on Monday, Tuesday, and Wednesday, February 15th, 16th, and 17th. H.R.H. the Duchess of Albany will open the bazaar on Monday, the ceremony being performed on the two following days by the Countess of Pembroke and the Dowager Countess De La Warr. An excellent and varied programme of entertainments will be carried out during the afternoons and evenings, at which many well-known theatrical and musical artists have generously offered to assist. Admission to bazaar from 2s. 6d. to 6d.; season ticket, 3s.

### Presentations.

On Saturday last, Miss H. Larsen, who has resigned the office of Matron to the North-Western Hospital, was presented by the medical superintendent, on behalf of the staff, with a fitted plate-chest and sundry articles for table use as "a mark of their esteem and regard." Miss Larsen, who has been in failing health for some time past, leaves England shortly for Denmark, her native land, carrying with her every good wish from those with whom she has worked so successfully and harmoniously.

### Wants and Workers.

Miss Holloway, hon. secretary South Oxon Nursing Association, Thame, will be glad if any reader of The Hospital can lend her the rules of some existing society for the Loan of Nursing Appliances, or give her advice in drawing up regulations as to the usual charge, and the things required for lending or for use in a small nursing home in a country town.

### Notes and Queries.

The contents of the Editor's Letter-box have now reached such unwieldy proportions that it has become necessary to establish a hard and fast rule regarding Answers to Correspondents. In future, all questions requiring replies will continue to be answered in this column without any fee. If an answer is required by letter, a fee of half-a-crown must be enclosed with the note containing the enquiry. We are always pleased to help our numerous correspondents to the fullest extent, and we can trust them to sympathise in the overwhelming amount of writing which makes the new rules a necessity. Every communication must be accompanied by the writer's name and address, otherwise it will receive no attention.

*Drink and Accidents.*

(127) Can you tell me the number of The Hospital in which the proportion of accidents occurring from drink is discussed? I think it must be about two years ago.—*Sister A.*

There was a leading article on this subject in The Hospital for April 6th, 1895, and a statement by the Hon. Sydney Holland in reply, the following week. The two numbers are being forwarded to you as you request.

*Uniforms.*

(128) Can you tell me (1) what are the outdoor uniforms of King's College Hospital and University College Hospital? and (2) Having suffered from slight spinal curvature and flat foot, could one, when cured, train as a nurse?

(1) You should address your inquiry to the hospitals in question. (2) Surely no one who knows what nursing is could imagine that a candidate with such physical weaknesses would be accepted at any hospital as a probationer! Very good health is an essential qualification.

*Barrow-in-Furness.*

(129) Are there any nursing homes in Barrow-in-Furness, or near that town?—*Inquirer.*

Do you mean an institution which sends out nurses, or a private home where paying patients are received? We do not know of either in Barrow-in-Furness or anywhere very near. You could best judge for yourself by consulting "Hospitals and Charities" (Scientific Press, 28 & 29, Southampton Street, Strand, London, W.C.), where are complete lists of such institutions. Perhaps some of our readers can give information to our correspondent.

*Nurses' Badges.*

(130) Can you give me the address for registration of nurses' badges?—*Enquirer.*

Please explain more clearly what you mean. What badges and what registration?

*Training.*

(131) Please advise me. I have had thorough training in fever nursing, and want general training, but do not think I should be strong enough, having a weak knee, to stand three years in hospital. Could I get a certificate anywhere for one year's training in general nursing? I cannot pay for it.—*F. T. C.*

Some of the smaller provincial hospitals give certificates for one year, but usually a premium is required. You could not get one year's training in any large general hospital without paying for it. If you want a certificate which would be really useful to you in the future, you will have to take two years' training at least; that is given at a good many provincial hospitals. Consult the lists in "How to Become a Nurse" (Scientific Press, 28 & 29, Southampton Street, Strand, London, W.C.), and apply to the matrons of those institutions which take probationers for one or two years.

(132) Would it be possible for a nurse to obtain one year's further hospital experience in a London hospital without premium, or where a very small one is required, after having had two years' training in a provincial hospital and two years' private nursing?—*Greta.*

You would not get appointed staff nurse in a general London hospital, these large institutions almost invariably promoting their own nurses; and in any case where an outsider might be eligible a three years' certificate would be required. You might get a post as staff nurse in a special hospital or at an infirmary. Your best plan will be to write direct to the matrons of some of the smaller London hospitals and of the Poor Law Infirmaries. Have you asked the advice of the matron of your own training school?

*Workhouse Infirmaries.*

(133) Can you tell me the number of beds in the Brownlow Hill Infirmary, Liverpool, and in the Crumpsall Infirmary, Manchester, and if there is any other infirmary with a greater number of beds than either of these?—*E.L. and C. K.*

Brownlow Hill Infirmary has about 1,700 beds; Birmingham Infirmary, 1,540 beds: Crumpsall, 1,350 beds. Other large infirmaries, which are also nurse training schools, are Mill Road Infirmary, Liverpool, with 850 beds; Salford and West Derby Union Infirmaries, each with 800 beds. In the forthcoming edition of Burdett's "Hospitals and Charities" particulars of these principal provincial infirmaries will be added to the Metropolitan ones.

*Probationers' Duties.*

(134) Can you recommend me a book on the duties of a probationer? And are daily probationers ever received at hospitals?—*L. T. G.*

Read "How to become a Nurse" (Scientific Press, 28 & 29, Southampton Street, Strand, London, W.C.), and for a nursing text book get Dr. Percy Lewis's "Theory and Practice of Nursing," from the same address. We have only heard of one country hospital where paying probationers were allowed to go by the day, and should hope it is not a plan which obtains at many institutions. If you want to learn nursing, enter a good training school for a term of three years' training.

